# Supplementary material for: Research and validation of forest carbon sequestration measurement model based on biomass method-A case study of Guizhou Province
Source: PLoS One. 2025 Apr 21;20(4):e0314405. doi: 10.1371/journal.pone.0314405 (PMC12011259; doi:10.1371/journal.pone.0314405)
Supplement: Attachment — Submitted filename: Response to Reviewers10.14.doc [file pone.0314405.s002.doc]

Dear editor：

Thank you very much for your letter. We have learned much from your and two reviewers’ comments, which are fair, encouraging and constructive. After carefully studying the comments and your advice, we have made corresponding changes. The main revisions are listed below.

# For reviewer one:

At present, there is no clear specification for the measurement method of forest carbon sequestration. The author chooses the biomass method, and its accuracy and principle need to be explained in the text. **Thanks to the opinions of the reviewer, This suggestion is very good and scientific. Indeed, we need to supplement the field data to verify the accuracy of the model. We have several field observation points in the field. I added the measured data of two points to verify the model. The article also added a corresponding chapter: Calculation and Model Validation of Carbon Sequestration in Field Monitoring Area.**

Calculation and Model Validation of Carbon Sequestration in Field Monitoring Area

In order to verify the accuracy of the model, we calculated the data of two sample plots monitored in the field of ecological restoration and compared them with the predicted data of the model.

**Basic data collection**

The data in this paper mainly come from the combination of field survey and data collection, through the field survey to obtain the tree height, diameter at breast height, crown width, height under branch, phenological period and viability of the arbors in the sample plot, and through the statistical yearbook, local chronicles and relevant literature of the project area to collect the local natural environment, natural resources, social and economic conditions and other information.

In this paper, the monitoring study plots are located in Majiatian Coal Mine and fei Longhu Wetland Park(Yu Qin), and two fixed plots are established for long-term monitoring. The construction and division of sample plots shall be carried out according to the technical specifications of the Center for Tropical Forest Science.

At the same time of establishing the sample plot, each tree species was numbered in the 10 m × 10 m sample plot. The number of the tree species was determined by the row number and column number of the tree species in the sample plot. The lower left corner of the sample plot was taken as the origin to arrange the column number and row number in the sample plot in the order of the coordinate axis. It is worth noting that the number first indicates the position of the column, and then indicates the position of the row. The individual numbering method of the tree species is as follows: firstly, English letters representing the location of the sample plot and Roman numerals representing the serial number of the waste dump are used together to represent the precise location of the sample plot, four-digit Arabic numerals are used to represent the location of the small sample in the sample plot, and a three-digit Arabic numeral is used to represent the specific tree number of the tree species.

In the 10 m×10 m quadrats, the individuals with the diameter at breast height (DBH) ≥ 1 cm were marked clockwise from the lower left corner. For trees with branches, the branches should also be labeled, for individual trunks or branches with DBH less than 5 cm, the label should be fixed with fishing line, for individual trees with DBH ≥ 5 cm, the label should be fixed with stainless steel nails, the height of the label from the ground is about 1.5 m, and the angle of the steel nails is about 60 degrees with the trunk obliquely upward. The depth of nailing into the trunk is about 1 cm, and the stumps for various reasons are marked. This verification only includes Majiatian Coal Mine (No.1 sample plot) and Yuqingfei Longhu Wetland Park (No.2 sample plot).

The DBH of tree species with DBH less than 5 cm shall be measured with vernier caliper, and the DBH of tree species with DBH greater than 5 cm shall be measured with DBH ruler; if there are branches below the DBH of tree species and the DBH of branches is greater than 1 cm, the DBH of branches shall be measured; When the tree height of some tree species in the sample plot is less than 1. 3 m, the diameter at breast height at 1. 3 m cannot be measured, so the vernier caliper is used to measure the base diameter, and the steel tape is used to measure and record the coordinate position in the 5 m × 5 m small quadrat. In the process of field survey, besides measuring the diameter at breast height of trees, it is also necessary to accurately record the name of the investigated tree species, tree number (tag), species name (species), coordinates (GX), tree height, crown width, phenological period and vitality, and to establish a sample tree species database.

The input of survey data adopts the dual-track method of letting two people input the same data, and checks the input results to ensure the correctness of data input. Use the relevant software Excel compare to compare the input results of the two people, the software will prompt the difference between the two input results, and then check the data to correct the wrong data. In order to facilitate the sharing of sample survey data and the processing of data, the database is standardized according to the standard requirements of CTFS, and the standardized database is Excel file.

**Standing wood volume analysis of each plot**

The total volume in plot 1 was 270 m^3^ , and the volume density was 32. 61 m^3^ · hm ^-2^ . The difference between the total volume of surviving individuals and the total volume of trees in the plot was only 0. 67 m^3^ , the proportion of the total volume of surviving individuals to the total volume of trees was 97. 26%, and the density of surviving volume was 29. 68 m^3^ · hm^-2^ .

The purpose of data unification is to unify the format and name fields of the data to be analyzed, such as the unification of different measurement units and species information. For the unification of measurement units, the professional forestry involved in this study, such as the three parameters of biomass, diameter at breast height and tree height, are measured in kg, trees per hectare, initial planting density per hectare, scattered tree species, growth and other survey factors. Human activity had little impact in the selected study area and growth was stable over time. Exclude special circumstances such as forestry activities, fruit tree planting and being in the urban green belt in the study area. Finally, the collected data is divided into two parts, which are used for training and simulation.(Table 3-4)

Table 3 Volume of Standing Timber in No.1 Sample Plot

| Sample group number | Timber volume of Robinia pseudoacacia | Number of Robinia pseudoacacia | Volume of Pinus tabulaeformis | Number of Pinus tabulaeformis | Volume of elm trees | Number of elm trees |
| --- | --- | --- | --- | --- | --- | --- |
|  |  |  |  |  |  |  |
| 1 | 3.82 | 156 | 0.31 | 38 | 5.54 | 19 |
| 2 | 1.82 | 121 | 0.23 | 36 | 5.72 | 19 |
| 3 | 2.41 | 122 | 0.26 | 38 | 8.29 | 21 |
| 4 | 2.43 | 96 | 0.19 | 32 | 2.78 | 19 |
| 5 | 2.31 | 86 | 0.13 | 36 | 19.98 | 24 |
| 6 | 2.51 | 95 | 0.2 | 35 | 6.46 | 19 |
| 7 | 2.46 | 103 | 0.18 | 33 | 24.94 | 23 |
| 8 | 1.82 | 90 | 0.22 | 37 | 17.09 | 26 |
| 9 | 2.21 | 105 | 0.23 | 34 | 7.94 | 22 |
| 10 | 1.35 | 62 | 0.19 | 34 | 12.92 | 17 |

Table 4 Table of Living Wood Volume of No.1 Sample Plot

| Sample group number | Timber volume of Robinia pseudoacacia | Number of Robinia pseudoacacia | Volume of Pinus tabulaeformis | Number of Pinus tabulaeformis |
| --- | --- | --- | --- | --- |
|  |  |  |  |  |
| 1 | 4.91 | 71 | 0.46 | 34 |
| 2 | 3.08 | 71 | 0.38 | 32 |
| 3 | 3.25 | 70 | 0.41 | 34 |
| 4 | 3.61 | 74 | 0.32 | 28 |
| 5 | 3.69 | 85 | 0.28 | 32 |
| 6 | 3.31 | 74 | 0.35 | 31 |
| 7 | 3.58 | 73 | 0.33 | 29 |
| 8 | 2.87 | 69 | 0.37 | 32 |
| 9 | 3.38 | 72 | 0.38 | 30 |
| 10 | 2.43 | 53 | 0.34 | 30 |

The total volume in plot 2 was 36. 00 m 3 , and the volume density was 45. 00 m 3 · hm -2 . The total volume of surviving trees was 30. 30 m 3 , which was 5. 70 m higher than total volume of trees 3 , and the density of surviving trees was 37. 88 m 3 · hm -2 . The volume data in the sample plot is shown in the following Table5-6.

| Sample groupnumber | Timber volume of Robinia pseudoacacia  （m^3^） | Number of Robinia pseudoacacia | Volume of Pinus tabulaeformis  （m^3^） | Number of Pinus tabulaeformis | Volume of elm trees  （m^3^） | Number of elm trees |
| --- | --- | --- | --- | --- | --- | --- |
| 1 | 3.63 | 128 | 0.25 | 23 | 0.59 | 51 |
| 2 | 3.45 | 111 | 0.22 | 15 | 0.86 | 43 |
| 3 | 2.45 | 95 | 0.36 | 19 | 0.61 | 29 |
| 4 | 3.06 | 120 | 0.18 | 16 | 0.33 | 39 |
| 5 | 3.38 | 136 | 0.11 | 13 | 0.11 | 37 |
| 6 | 3.04 | 129 | 0.23 | 16 | 0.40 | 49 |
| 7 | 3.10 | 121 | 0.25 | 21 | 0.34 | 47 |
| 8 | 2.99 | 121 | 0.18 | 16 | 0.24 | 34 |
| 9 | 3.10 | 130 | 0.30 | 16 | 0.14 | 49 |
| 10 | 2.50 | 124 | 0.24 | 15 | 0.56 | 45 |

Table 6	Table of Living Wood Volume of No.2 Sample Plot

| Sample group number | Timber volume of Robinia pseudoacacia  （m^3^） | Number of Robinia pseudoacacia | Volume of Pinus tabulaeformis  （m^3^） | Number of Pinus tabulaeformis |
| --- | --- | --- | --- | --- |
| 1 | 3.51 | 92 | 0.24 | 20 |
| 2 | 2.61 | 72 | 0.19 | 14 |
| 3 | 2.45 | 95 | 0.36 | 18 |
| 4 | 2.89 | 83 | 0.17 | 15 |
| 5 | 3.22 | 81 | 0.10 | 11 |
| 6 | 2.52 | 74 | 0.23 | 16 |
| 7 | 2.94 | 86 | 0.25 | 20 |
| 8 | 2.82 | 77 | 0.18 | 16 |
| 9 | 2.92 | 91 | 0.29 | 16 |
| 10 | 2.16 | 71 | 0.23 | 15 |
| Subtotal | 28.05 | 822 | 2.25 | 161 |

Forest stock volume refers to the total volume of existing trees in a certain area of forest. It generally refers to the volume of the trunk including bark. Sometimes, in order to get more accurate results, it is calculated separately according to tree species, diameter class, timber species, etc. The unit of forest stock volume is cubic meter. In this paper, the forest volume is calculated separately according to the tree species in the same plot. The final calculation results are shown in the following table, and the total volume of each tree species and the average volume of a single tree are compared and analyzed.(Table 7)

Table 7 Forest Volume in Each Plot

| Sample plot information | Plot 1 | Plot 2 |
| --- | --- | --- |
| Forest stock |  |  |
| （m^3^） | 24.41 | 36.00 |
| Volume of surviving wood |  |  |
| （m^3^） | 23.74 | 30.30 |
| Stand volume density |  |  |
| （m^3^/hm^2^） | 30.51 | 45.00 |

Based on the measured data, the results showed that the biomass of plot 1 was the highest, reaching 55. 08 t (for the convenience of comparison, the area of plot was calculated as 1. 0 hm 2 , the same below). The carbon sink density is 21.24 t · hm -2 . The carbon sink of plot 2 was 21. 65 t, and the biomass of plot 1 was 56. 58 t, and the carbon sink of plot 2 was 22. 26 t. The results show that the average relative error is less than 2.5% and the absolute error ranges from 0.61 to 1.5t/hm ^2^ , which verifies the reliability of the model.

1. This paper lacks a discussion of the results of the derivation of the formula, which I believe is a major flaw, a valuable literature should be discussed in depth in the discussion section to discuss the contribution of this study, and the results of previous research results are compared to illustrate the strengths and weaknesses of the results of the study.

#  Thanks to the opinions of the reviewer, This suggestion is very good and scientific.In the last conclusion, we add the corresponding content in the discussion part.

Since the 1990s, there have been many studies on forest biomass abroad. Ter-Mi-kaelian

& korzukhin [17]reviewed the modeling equations for standing tree biomass of 65 tree species in North America. A total of 803 modeling equations of the form M = aD^b^ were given. Parresol[18] reviewed the biomass models and the methods of weighted analysis, discussed not only the problems of additivity and coordination, the error components of stand biomass estimation, but also the evaluation of biomass model indices. Biomass equations of six tree species in the northern forests of Manitoba, Canada, were established by Bond Lamberty et al[19].Snorrason & Einarsson[20] established the stem volume equation and aboveground biomass equation of 11 main tree species in Iceland. The total above-ground volume of seven main tree species in France was established Vallet et al.[21] Biomass models were developed Basuki et al[22]for four Dipterocarpaceae genera in forests in the eastern region of Galinmandan, Indonesia. N Návar developed the relative growth equations for foliage, trunk, biomass, and stem biomass for 10 tree species in the temperate and tropical forests of northwestern Mexico. The study of forest biomass in China started late[23-30]. Since the 1990s, the biomass of Pinus massoniana in southern China has been studied, and the compatible tree biomass model compatible with volume has been established by the method of linear simultaneous equations. Taking Cunninghamia lanceolata in southern China as the research object, Zeng Weisheng et al established a nonlinear biomass model system, which not only considered the compatibility of biomass and volume, but also satisfied that the total biomass of trees was equal to the sum of all components. Xu Hui studied the biomass of Larix gmelinii, Tilia amurensis and Abies faxoniana in northern China, and established the models of biomass of each part and total biomass compatible with volume. Zhang Huiru studied the biomass of Tilia amurensis and Larix gmelinii, and established the nonlinear models of total biomass and biomass of each part with volume, diameter, tree height, crown width, crown length and other factors. Taking Larix gmelinii in Northeast China as the research object, Tang Shouzheng made a comparative analysis and evaluation of three biomass estimation methods and two proportional adjustment methods. Different experimental results show that the prediction of belowground biomass and aboveground biomass is very close to the expected results, but the prediction of belowground biomass is still not ideal[31-35]. Biomass evaluation index pairs of different models are shown in Table 8 and Table 9.

Table 8 Comparison of aboveground biomass evaluation indexes of different models

| Model | Evaluation index | | | |
| --- | --- | --- | --- | --- |
|  | R^2^ | MAE | RMSE | MSE |
| Traditional binary regression model | 0.8102 | - | - | - |
| BP neural network model | 0.927 | 15.6935 | 24.8975 | 619.8871 |
| Neural Network Model after Replacing sigmoid Function with reLU | 0.9702 | 9.2669 | 15.9194 | 253.4283 |
| Neural network model after replacing MSEloss with binary _ crossentropy; | 0.9829 | 6.5266 | 12.0536 | 145.2886 |

Through the study, it is found that compared with the traditional binary regression model, the neural network is more accurate in estimating the determination coefficient R2 of biomass, while the MAE, RMSE and MSE index coefficients are getting smaller and smaller, indicating that the model fitting accuracy is better.

Table 9 Comparison of Underground Biomass Evaluation Indexes of Different Models

| Model | Evaluation index | | | |
| --- | --- | --- | --- | --- |
|  | R^2^ | MAE | RMSE | MSE |
| Traditional binary regression model | 0.8102 | - | - | - |
| BP neural network model | 0.927 | 15.6935 | 24.8975 | 619.8871 |
| Neural Network Model after Replacing sigmoid Function with reLU | 0.9702 | 9.2669 | 15.9194 | 253.4283 |
| Neural network model after replacing MSEloss with binary _ crossentropy; | 0.9829 | 6.5266 | 12.0536 | 145.2886 |

It can be seen from the results that all the indexes of the optimized neural network are close to the better parameters. The prediction accuracy of the model for the underground biomass of the tree species is greatly improved.

This paper mainly deduces an accurate and feasible carbon sequestration model based on biomass method. This paper first introduces the concept of carbon sink project boundary and how to determine the project boundary, and also introduces the selection and decision-making methods of carbon pool and greenhouse gas emissions. In the process of measurement, considering that it is impossible to calculate all the forest land, this simulation derives the corresponding sample plot sampling calculation formula and designs the sample plot on the premise of ensuring the accuracy requirements. Then the whole carbon sequestration model is constructed, in which sampling design, project boundary, carbon pool selection and other factors are considered comprehensively, and the actual net carbon sequestration caused by CH_4_ , N_2_O and other gas emissions in forestry carbon sequestration projects is not accurate. Combined with the actual project development needs, the specific calculation model is derived. Finally, according to the strong time series characteristics of key factors such as diameter at breast height and tree height in the process of forest growth, taking Cunninghamia lanceolata as an example, the forestry carbon sequestration measurement model in this chapter was verified by using this characteristic, the average relative error was 6.09%, and the absolute error range was 0.348-4.262/hm 2 , thus the reliability of the model was verified.

3.Lines 50-55: Lack of source references for data listed in the introduction.The algorithms used in the study and the implementation steps are explained in detail. The result of the study showed that the RF-RFE algorithm produced consistent results.

. Thanks to the opinions of the reviewer, .We have made corresponding modifications

Data source: Official website of Guizhou Forestry Bureau

4.Lines 57-58: In the introduction, it is mentioned that some traditional research methods have the problems of heavy workload and too much reliance on manual labor, which research methods are specifically referred to, and please give examples of traditional methods in the existing literature to provide an in-depth compendium and summarize the progress.

. Thanks to the opinions of the reviewer, .We have made corresponding modifications

We explained and organized these methods during the conclusion discussion

5.Line 76: Is the capital letter “O” a misprint? Or does it have a specific meaning? This is difficult to understand, so please clarify.

. Thanks to the opinions of the reviewer, .I'm very sorry, this was our mistakes We have made corresponding modifications

We deleted it6.Lines 236-240, the topic of the article as well as the entire text talks about forest carbon sinks, so why does the sampling design jump to agricultural carbon sinks? This is a basic logical flaw.

. Thanks to the opinions of the reviewer, .I'm very sorry, this was our mistakes We have made corresponding modifications

Corrected as:Forestry carbon

7.Lines 435-437 state that this paper derives a formula for sample plot sampling, which, as far as I know, is derived from the methodology for afforestation carbon sink projects (CCER-14-001-V01), what is the difference between the two? Please clarify.

8.the text of a large part of the reference afforestation carbon sink project methodology (CCER-14-001-V01) to do a lot of qualitative description, if as a carbon sink development project it is necessary, but for a scientific research theme, I think that padding is more redundant.

The reviewers are very professional, which is admirable,This is a matter of ownership, to demonstrate that the government can profit from it and generate income,This document (CCER-14-001-V01)applies to afforestation of trees, bamboo, and shrubs, including protective forests, special-purpose forests, timber forests, etc. It does not include afforestation of economic forests, greening of non forest roads, greening of urban villages and industrial and mining land. The afforestation carbon sink projects using this document must meet the following conditions:

a) At least three years prior to the start of the project, the project land shall be planned as forest land that does not meet the definition of forest;

b) The land ownership of the project is clear, with real estate ownership certificates, land contracting or transfer contracts; Or land certificates or forest rights certificates approved and issued by the people's government or competent department with approval authority;

c) The continuous area of a single plot of land in the project shall not be less than 400m2. For projects starting before 2019 (inclusive), the continuous land area shall not be less than 667m2;

d) The project land does not belong to wetlands;

e) The project will not remove existing scattered trees and bamboo, and the total removal ratio of existing shrubs and bamboo with a diameter at breast height less than 2cm shall not exceed 20% of the land surface area within the project boundary;

f) Except for land preparation and afforestation at the beginning of the project, no repeated disturbance of the soil shall be carried out during the accounting period;

g) Except for necessary burning of infected trees, the project does not allow other people to burn them;

h) The project will not cause the transfer of agricultural activities (such as planting, grazing, etc.) within the project boundary, that is, there will be no leakage;

i) The project should comply with legal and regulatory requirements, as well as industry development policies.This government can solve it from a policy perspective, and we are working on this for the Guizhou Provincial Department of Natural Resources

9. The article has fewer references (only 16), which is a side reflection of the inadequacy of this paper to sort out the current status and progress of research.

Thanks to the opinions of the reviewer, .We have made corresponding modifications,If you have good article recommendations, please let us know, we also need to keep learning

# For reviewer two:

1 At present, there is no clear specification for the measurement method of forest carbon sequestration. The author chooses the biomass method, and its accuracy and principle need to be explained in the text.

This is a great question, and we have added it to the discussion section in the article

2 There is a forestry carbon sequestration measurement and prediction system or platform mentioned in the article, but it is not actually pointed out. The author needs to add a case to illustrate that forestry carbon sequestration has been recognized to some extent.

Due to the fact that forestry carbon sequestration measurement and prediction systems or platforms are currently managed by the government, many data have authority, which is not convenient to display in the article. Reviewers can log in to the Guizhou Ecological Product Registration and Evaluation System (gzstqy. com), which is a screenshot of the website

3 Under the baseline scenario, the baseline carbon layer change can be regarded as 0. Why?

It can be seen that the reviewer is very professional and immediately asked the core technical questions

This is a method of operation for the project, aimed at avoiding interference and ownership disputes. In the baseline scenario, due to the pre cleaning of the project site, some projects may have no debris before development, and the change in ∆𝐶𝐵𝑆𝐿 of the baseline carbon layer can be considered as 0.4 Most of the sample plots set by the project are rectangular or square, but the forest on the spot can not be so regular, how to eliminate such errors?

Great question, the proportion of carbon sink in different terrestrial ecosystems varies. For example, the biomass on artificial forests is higher than that on farmland, and the carbon storage in soil carbon pools in grassland land use systems is huge. Therefore, in the process of selecting carbon pools, it is necessary to consider the land use system comprehensively before making a decision. The carbon pool of litter or dead wood is basically non-existent in grassland systems. For all forestry carbon sinks, above ground organisms.The carbon pool is the most important and must be selected during the measurement process, while other carbon pools can be selected based on taking into account the actual development situation, the article lists some types of carbon pools for selection.

Based on the actual development and comprehensive consideration, select a carbon pool. The fewer carbon pools selected, the lower the measurement cost. Finally,the greater the benefits obtained, the more homeowners will be attracted to invest in carbon sequestration development, which can not only improve high project development enthusiasm and economic value generation can also contribute to the healthy and rapid development of China's low-carbon economy exhibition. Therefore, in the actual development process, the measurement cost of the entire project is much lower than the carbon sink generated by the project Value is the key to ensuring the smooth progress of the project, so some carbon stocks can be neglected for measurement during development.In projects that do not consider costs, neglecting the increase in carbon storage leads to an increase in project costs The problem of inaccurate carbon sequestration measurement in actual projects is not in line with project requirements. By combining In actual development situations, there are some projects that do not need to ignore carbon storage, so we can select carbon banks based on actual project requirements.

5 Most of the sample plots set by the project are rectangular or square, but the forest on the spot can not be so regular, how to eliminate such errors?

This is why we have parameter errors. Generally, we use weighted averages to reduce this error. However, it cannot be denied that this is a problem that must be faced, and there may be discrepancies between simulation and actual situations

6 The English expression of the article needs to be strengthened

Yes, this is our shortcoming. We have contacted the official polishing agency and will conduct professional polishing after the professional issues are corrected

Yang Wu
