# Supplementary material for: Research and validation of forest carbon sequestration measurement model based on biomass method-A case study of Guizhou Province
Source: PLoS One. 2025 Apr 21;20(4):e0314405. doi: 10.1371/journal.pone.0314405 (PMC12011259; doi:10.1371/journal.pone.0314405)
Supplement: Attachment — Submitted filename: Response to Reviewers11.1.doc [file pone.0314405.s003.doc]

# For reviewer one:

1. Regarding the fourth review comment, the author did not understand my meaning. What I mean is that the author needs to organize in the introduction rather than just in the discussion.
   “4. Lines 57-58: In the introduction, it is mentioned that some traditional research methods have the problems of heavy workload and too much reliance on manual labor, which research methods are specifically referred to, and please give examples of traditional methods in the existing literature to provide an in-depth compendium and summarize the progress.”

**I am sorry that the author did not understand the suggestions of the reviewer at the beginning, and we have supplemented the revision. The traditional method is time-consuming and labor-intensive, and the representative methods are the reserve change method and the profit and loss method. The reserve change method requires that the two survey data are obtained by using the same method, using the same biomass model and parameters, and the data based on continuous sample plots are the most accurate and reliable, but the workload is large, and the accuracy can only be guaranteed on the scale of the sampling population. The profit and loss method obtains the forest carbon sink through the empirical statistical model and the process model, but it has strong regional characteristics, so it needs to re-determine the empirical parameter values or the localized process model parameters when it is applied in other regions, and the parameters of the process model are usually not easy to obtain, which limits the use of the model.**

**REVISION:**At present, the measurement problems encountered in the development of forestry carbon sequestration are still in the research stage. Some traditional research methods have the problems of heavy workload and over-reliance on manual work, such as the representative reserve change method and profit and loss method [6]. The change in reserves method evaluates the annual change in carbon stocks over time [7]. This method obtains basic data, mainly forest volume, through a large number of ground surveys (such as the National Forest Inventory)[8]. Then the carbon storage of each carbon pool of the ecosystem is estimated by using the established biomass model and carbon measurement parameters (forestry industry standards or related literature), or related equations, biomass carbon density and soil organic carbon density, etc. Finally, the annual change of carbon storage in a period of time is analyzed. If the annual variation of carbon storage is positive, it is regarded as a "carbon sink", otherwise, it is regarded as a "carbon source". Up to 2023, China has carried out several continuous forest resource inventories at intervals of 5 years, and many provinces and regions have also carried out the second class forest resource inventory for 10 years. These survey data and statistical data are the basis of this method.The profit and loss method is to assess the carbon sink function of the ecosystem by studying the carbon budget of the ecosystem in a certain period of time and using the balance results of carbon absorption and emission. This requires a lot of manpower to measure the biomass in the field and establish a regression model with DBH, tree height, volume, forest age and other variables to describe the growth rate. This method can also be used to assess and predict the impact of climate and other environmental factors on growth and carbon accumulation. Commonly used models include FVS [9,10], CACTOS [11], ORGANON [12] and TreeGross [13]. Full carbon pool models mainly include CO2FIX [14], TreeGroSS-C [15], FORCARB [16] and CBM-CFS3 [17-18].The reserve change method requires that the two survey data are obtained by using the same method, using the same biomass model and parameters, and the data based on continuous sample plots are the most accurate and reliable, but the workload is large, and the accuracy can only be guaranteed on the scale of the sampling population. The profit and loss method obtains the forest carbon sink through the empirical statistical model and the process model, but it has strong regional characteristics, so it needs to re-determine the empirical parameter values or the localized process model parameters when it is applied in other regions, and the parameters of the process model are usually not easy to obtain, which limits the use of the model. On the other hand, there is no clear specification for the measurement methods of forest carbon sinks at present. The existing measurement methods mainly include biomass method, volume method and eddy correlation method. Among these methods, the biomass method has high practicability because of its simple and convenient operation, so this paper mainly studies the application of biomass method in the theoretical study of forestry carbon sequestration.

**We have also added relevant literature. If the reviewer has any good literature recommendations, please let us know and we will continue to learn**

#2. The author seems to have omitted a response to the seventh review comment: The specific review comments are as follows:“7.Lines 435-437 state that this paper derives a formula for sample plot sampling, which, as far as I know, is derived from the methodology for afforestation carbon sink projects (CCER-14-001-V01), what is the difference between the two? Please clarify.

#  There is no difference. We use the formula in (CCER-14-001-V01) for reference. We are very sorry that there is no reference. We have made corrections.(reference 21) Thank you again for the professional advice of the reviewer, which is very helpful to us.

# For reviewer two:

**Thank you very much for your review and suggestions. Wishing you a happy life and smooth work,We have made corresponding modifications to your suggestions in the text**
